# Early evolution of the gular musculature and its innervation in ray‐finned fishes

**DOI:** 10.1002/ar.70204

**Published:** 2026-04-06

**Authors:** Aléssio Datovo, Paulo Presti, George Vita, Murilo N. L. Pastana, Luiz A. W. Peixoto

**Affiliations:** ^1^ Museu de Zoologia da Universidade de São Paulo (MZUSP) São Paulo Brazil; ^2^ Instituto de Biociências da Universidade de São Paulo (IBUSP) São Paulo Brazil; ^3^ Coordenação de Biodiversidade Instituto Nacional de Pesquisas da Amazônia (INPA) Manaus Brazil

**Keywords:** actinopterygii, cranial muscles, evolutionary morphology, holostei, myology, teleostei

## Abstract

Gular muscles are an important but often overlooked component of cranial anatomy in bony fishes. They are located on the ventral surface of the head and are derived from the mandibular and hyoid arches. We present a comprehensive review of the gular musculature and its innervation across early diverging actinopterygian lineages. By integrating data from sarcopterygian and chondrichthyan outgroups, we refine existing homology hypotheses, resolve terminological inconsistencies, and uncover a series of previously unrecognized evolutionary innovations that occurred early in ray‐finned fish phylogeny. These include the origin of novel muscular subdivisions, the reconfiguration of attachments, and shifts in functional roles. Notably, while ancestral gular muscles functioned exclusively in buccopharyngeal compression, we show that in Neopterygii and Teleostei, parts of this muscle complex were co‐opted to also perform buccopharyngeal expansion. This was achieved primarily through the attachment of the interhyoideus to the lower jaw and its anterolateral repositioning relative to the hyoid bar. These transformations represented a significant functional shift toward the sophistication of suction feeding in teleosts. Our analyses also reveal distinct lineage‐specific patterns of motor innervation in the gular muscles of fishes: whereas in most cartilaginous fishes the facial nerve extends anteriorly to also innervate mandibular‐arch muscles, in bony fishes the trigeminal nerve typically extends posteriorly to also innervate hyoid muscles. These findings provide additional evidence against the widely held assumption of nerve‐muscle specificity.

## 1 INTRODUCTION

The gular musculature in bony fishes comprises the superficial muscles at the ventral surface of the head. These muscles derive ontogenetically from the ventral portion of the mandibular and hyoid muscle plates (Edgeworth, 1935). Despite their superficial disposition on the head, dissection of the gular muscle can be challenging. In most cases, the subdermal connective tissue firmly adheres to the muscular surface, making it difficult to remove the skin without damaging the underlying muscles. This is more complicated in laterally compressed fishes, which typically have extremely thin hyoid muscles (e.g., osteoglossids, clupeiforms, stomiiforms). These obstacles probably explain why gular muscles in actinopterygians have been so poorly studied. In addition, these muscles show different patterns of innervation in distinct groups of jawed fishes (Holmqvist, 1911; Luther, 1913). As a result, the terminology applied to gular muscles in the literature varies widely (cf., Winterbottom, 1974), and little is known about their homologies, function, and phylogenetic implications.

In the present paper, we revised the gular musculature and its innervation in basal actinopterygians, clarifying homologies and standardizing anatomical terminology. Comparisons with agnathans, sarcopterygians, and cartilaginous fishes revealed several evolutionary novelties of the gular musculature that characterize the early nodes of the actinopterygian tree. Finally, we discuss the role of innervation in inferring muscle homologies and explore the functional implications of the changes observed in the gular muscles of basal actinopterygians.

## 2 MATERIALS AND METHODS

### 2.1 Specimens

This study was carried out with approval from the Animal Care and Use Committee (ACUC) of the Institute of Biosciences, University of São Paulo to A. Datovo (Project #226/2015; CIAEP #01.0165.2014). The research employed only ethanol‐preserved specimens, deposited in museums, and did not involve animal experimentation or fossil examination. Examined material is listed in Table 1 and is deposited in the following institutions: American Museum of Natural History, USA (AMNH); Field Museum of Natural History, USA (FMNH); Laboratório de Biologia e Genética de Peixes, Universidade Estadual Júlio de Mesquita Filho, Brazil (LBP); Laboratório de Ictiologia de Ribeirão Preto, Universidade de São Paulo, Brazil (LIRP); Museu de Zoologia da Universidade de São Paulo, Brazil (MZUSP); National Museum of Natural History, Smithsonian Institution, USA (USNM); Virginia Institute of Marine Science, USA (VIMS). Access to the material of those collections and permission for dissections were authorized by the respective curators.

**TABLE 1:** Material examined.

Order	Family	Species	Catalog #	*N*
Acipenseriformes	Acipenseridae	*Acipenser fulvescens*	MZUSP 48364	1
		*Huso huso*	USNM 309302	1
	Polyodontidae	*Polyodon spathula*	USNM 32387	1
Alepocephaliformes	Alepocephalidae	*Talismania homoptera*	USNM 410566	1
		*Xenodermichthys copei*	MZUSP 86570	1
Albuliformes	Albulidae	*Albula vulpes*	MZUSP 10625	1
Amiiformes	Amiidae	*Amia calva*	USNM 64338	1
Argentiniformes	Argentinidae	*Argentina striata*	MZUSP 17914	1
Ateleopodiformes	Ateleopodidae	*Ijimaia antillarum*	USNM 157838	1
Aulopiformes	Aulopidae	*Hime japonica*	USNM 384078	1
	Synodontidae	*Saurida caribbaea*	MZUSP 10647	2
Ceratodontiformes	Lepidosirenidae	*Lepidosiren paradoxa*	MZUSP 104621	1
	Neoceratodontidae	*Neoceratodus forsteri*	FMNH 147289	1
			USNM 40011	1
			USNM 327317	1
	Protopteridae	*Protopterus annectens*	MZUSP 84530	1
Characiformes	Bryconidae	*Brycon orbignyanus*	LIRP 6003	2
	Citharinidae	*Citharinus latus*	MZUSP 84480	1
	Distichodontidae	*Xenocharax spilurus*	MZUSP 50358	1
Chimaeriformes	Callorhinchidae	*Callorynchus milli*	USNM 318390	1
	Chimaeridae	*Hydrolagus alberti*	USNM 222801	1
Clupeiformes	Denticeptidae	*Denticeps clupeoides*	MZUSP 84776	2
	Pristigasteridae	*Pellona harroweri*	MZUSP 11364	2
Coelacanthiformes	Latimeriidae	*Latimeria chalumnae*	FMNH 76057	1
Cypriniformes	Cyprinidae	*Danio rerio*	MZUSP 112354	3
		*Rasbora cephalotaenia*	USNM 330848	1
Echinorhiniformes	Echinorhinidae	*Echinorhinus cookei*	USNM 179805	1
Elopiformes	Elopidae	*Elops lacerta*	MZUSP 84787	1
	Megalopidae	*Megalops cyprinoides*	USNM 102685	1
Gonorynchiformes	Chanidae	*Chanos chanos*	USNM 173572	1
			USNM 347536	1
Heterodontiformes	Heterodontidae	*Heterodotnus francisci*	USNM 45507	1
Hexanchiformes	Chlamydoselachidae	*Chlamydoselachus anguineus*	USNM 396127	1
	Hexanchidae	*Notorynchus cepedianus*	USNM 27191	1
Hiodontiformes	Hiodontidae	*Hiodon tergisus*	USNM 167970	1
Lepisosteiformes	Lepisosteidae	*Atractosteus tropicus*	USNM 120715	1
		*Lepisosteus platostomus*	USNM 54983	1
Myctophiformes	Myctophidae	*Diaphus dumerilii*	MZUSP 90106	2
	Neoscopelidae	*Neoscopelus macrolepidotus*	USNM 301041	1
Notacanthiformes	Halosauridae	*Halosaurus pectoralis*	USNM 317567	1
Osmeriformes	Osmeridae	*Osmerus mordax*	USNM 395752	1
Osteoglossiformes	Arapaimidae	*Arapaima gigas*	MZUSP 26083	1
	Osteoglossidae	*Osteoglossum ferreirai*	USNM 300966	1
Petromyzontiformes	Petromyzontidae	*Lampetra apêndix*	USNM 100302	2
		*Petromyzon marinus*	USNM 193068	2
Polypteriformes	Polypteridae	*Polypterus delhezi*	USNM 191616	1
		*Polypterus senegalus*	USNM 229760	1
Salmoniformes	Esocidae	*Esox americanus*	USNM 237253	1
	Salmonidae	*Oncorhynchus mykiss*	MZUSP 85378	1
Siluriformes	Diplomystidae	*Diplomystes mesembrinus*	LBP 449	1
	Nematogenyidae	*Nematogenys inermis*	LBP 1002	1
Stomiiformes	Sternoptychidae	*Maurolicus stehmanni*	MZUSP 80266	2

*Note*: *N*, number of specimens dissected. Taxonomic classification follows Fricke et al. (2025).

### 2.2 Techniques

Preserved specimens were double‐stained for cartilage and bone prior to dissections following the methodology of Datovo and Bockmann (2010). Dissections were made under a stereomicroscope. Anatomical drawings were based on photographs and direct stereomicroscopic observations of specimens, allowing for the capture of fine anatomical details. Drawings are bidimensional and were all produced with a Wacom Intuos4 pen tablet (Wacom Company, Ltd., Tokyo, Japan). Outlines were generated in Adobe Illustrator CC, and shading and coloring were applied in Adobe Photoshop CC (Adobe Systems, San Jose, CA).

### 2.3 Anatomical terminology

In the anatomical descriptions, the term insertion refers to the attachment of the muscle to the structure (usually a bone) that presumably moves (or moves more intensely) during its contraction; origin is defined as the opposite muscle attachment to the stationary (or less movable) skeletal element (Winterbottom, 1974). Musculous attachment (origin or insertion) is when the muscle fibers attach directly to the skeleton without the mediation of any macroscopically evident tendon. In the tendinous attachment, the muscle fibers converge onto a macroscopically evident tendon, which, in turn, attaches to the skeleton. In some instances, the attachment of a muscle is partially musculous and partially tendinous.

### 2.4 Ancestral state reconstructions

Ancestral state reconstruction was performed using a Bayesian framework (Data S1, Supporting Information) implemented in the R packages *ape* v. 5.8.1 (Paradis & Schliep, 2019) and *phytools* v. 2.5.2 (Revell, 2012). Character states were coded as binary (0/1) for all terminal taxa. Species with inapplicable character states (coded as “‐”) were excluded from the analysis by pruning them from the phylogenetic tree, as these states do not represent biologically meaningful variation for the character under study. Species with unknown character states (coded as “?”) were retained in the phylogeny, and uncertainty was incorporated into the reconstruction through multiple stochastic character mapping (see below).

We fitted three continuous‐time Markov (Mk) models of discrete character evolution (Lewis, 2001): equal rates (ER), symmetric rates (SYM), and all rates different (ARD). Model selection was performed using the corrected Akaike Information Criterion (AICc; Hurvich & Tsai, 1989), with Akaike weights (AICw) calculated from taxa with known character states only, following standard likelihood‐based procedures (Burnham & Anderson, 2002). The model with the highest Akaike weight was selected as the best‐fitting model for subsequent ancestral state reconstruction.

Joint ancestral state estimation was conducted using stochastic character mapping (Huelsenbeck et al., 2003) via the make.simmap function, with 1000 simulations performed under the best‐fitting Mk model. For species with unknown character states, we implemented a multiple imputation approach: 500 simulations assumed state “0” and 500 simulations assumed state “1” for the unknown taxa, effectively integrating uncertainty across all possible character state assignments. The combined set of 1000 stochastic maps was summarized to obtain posterior probabilities for each ancestral node, representing the proportion of simulations reconstructing each state at that node. All analyses were conducted in R v. 4.4.2 (R Core Team, 2024). The time‐calibrated phylogeny employed in the analyses was extracted from the Timetree of Life (Kumar et al., 2022).

## 3 RESULTS

### 3.1 Anatomical descriptions and synonymy

We provide below anatomical descriptions of the gular musculature and its innervation across key examined taxa. A synonymy of the myological terminology adopted in major previous studies is available in Data S3.

#### 3.1.1 Polypteriformes (Figures 1 and 2)

**FIGURE 1:**
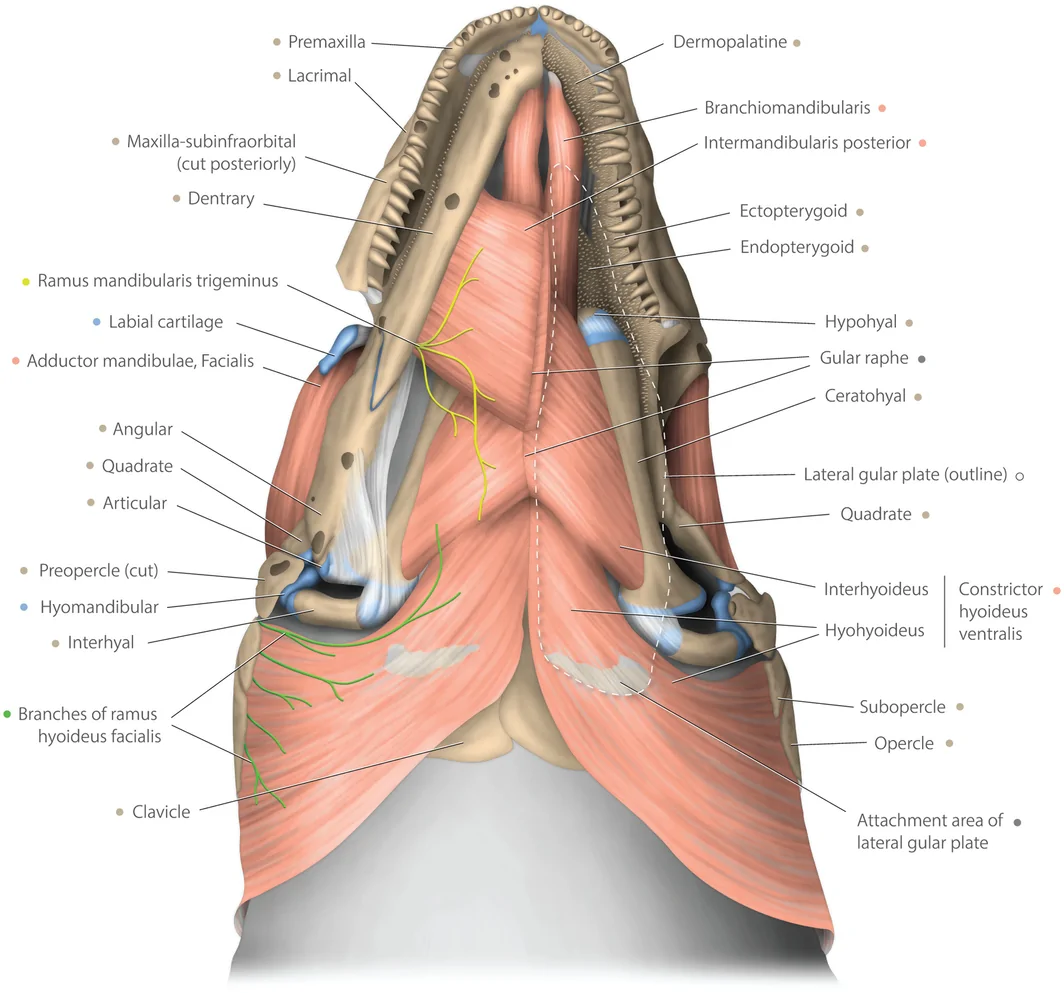
Ventral view of cranial musculoskeletal system and associated major motor branches of trigeminus and facialis nerves of *Polypterus senegalus* (USNM 229760, 108 mm SL). Lateral gular plates, quadratojugals, posterior maxillary segments, posterior portion of maxilla‐subinfraorbitals, and bony plates of preopercle and left lower jaw, adductor mandibulae, intermandibularis posterior, mandibulo‐hyoid ligaments, and nerves removed. Contour of right lateral gular plate represented by dashed line.

**FIGURE 2:**
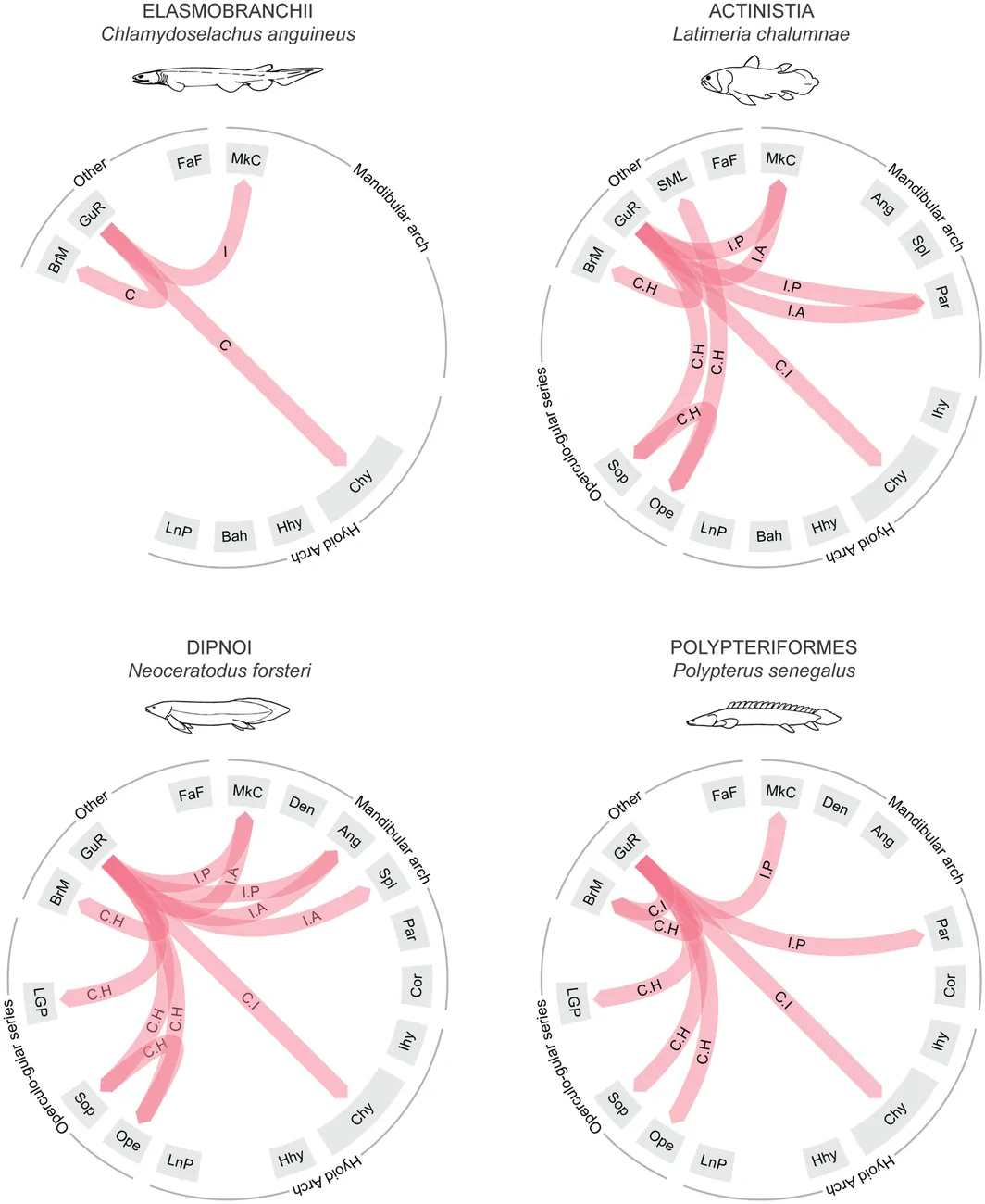
Connection diagrams of gular muscles in representatives of elasmobranch, actinistian, dipnoan, and cladistian fishes. Primarily compressive actions indicated in red. Straight and pointed tips indicate muscle origin and insertion, respectively. Ang, angular; Bah, basihyal; BrM, branchiostegal membrane; C, constrictor hyoideus ventralis; C.H, hyohyoideus; C.I, interhyoideus; Chy, ceratohyal; Cor, coronoid; Den, dentary; FaF, facial subdermal fascia; GuR, gular raphe; Hhy, hypohyal; I, intermandibularis; I.A, intermandibularis anterior; I.P, intermandibularis posterior; Ihy, interhyal; LGP, lateral gular plate; LnP, lingual pad; MkC, Meckel's cartilage; Ope, opercle; Par, prearticular; SML, suboperculo‐mandibular ligament; Sop, subopercle/subopercular cartilage(s); Spl, splenial.

The intermandibularis anterior is absent. The intermandibularis posterior is undivided and associated medially with a sagittal raphe, which is shared with the interhyoideus. We refer to this structure herein as the gular raphe. The intermandibularis posterior inserts along the ventral margin of the prearticular and Meckel's cartilage.

The interhyoideus originates primarily from the gular raphe shared with the intermandibularis; a few anterolateral fibers originate from the connective tissue lying ventral to the branchiomandibularis (=coracomandibularis). The insertion is mainly on the posteroventral region of the ceratohyal. A few posteriormost fibers merge distally with the hyohyoideus and insert on the branchiostegal membrane.

The hyohyoideus is undivided along its entire extent. The muscle originates from a tiny portion of the gular raphe and, most significantly, from the reinforced portions of a complex V‐shaped connective tissue formation at the confluence of the anteroventral end of the branchiostegal membrane and the walls of the sternohyoideus. We refer to this tendinous formation as the subhyoid tendon, and it lies dorsal to the interhyoideus. From this origin, the hyohyoideus radiates posterolaterally and is intimately associated with the entire connective tissue forming the branchiostegal membrane. In the anterior region of the muscle, several fibers attach to the posterior region of the lateral gular plate. Posterodorsally, the fibers of the hyohyoideus attach to the ventromedial aspects of the opercle and subopercle.

Branches of the ramus mandibularis trigeminus innervate the intermandibularis posterior and interhyoideus. Branches of ramus hyoideus facialis innervate the hyohyoideus and the interhyoideus. We observed one anastomosis on the right side of *P. delhezi* between different branches of the ramus mandibularis trigeminus. Still, we could not detect anastomoses between branches of that ramus with those of the ramus hyoideus facialis in any examined specimen.

#### 3.1.2 Acipenseriformes

##### Acipenseridae (Figures 3 and 4)

**FIGURE 3:**
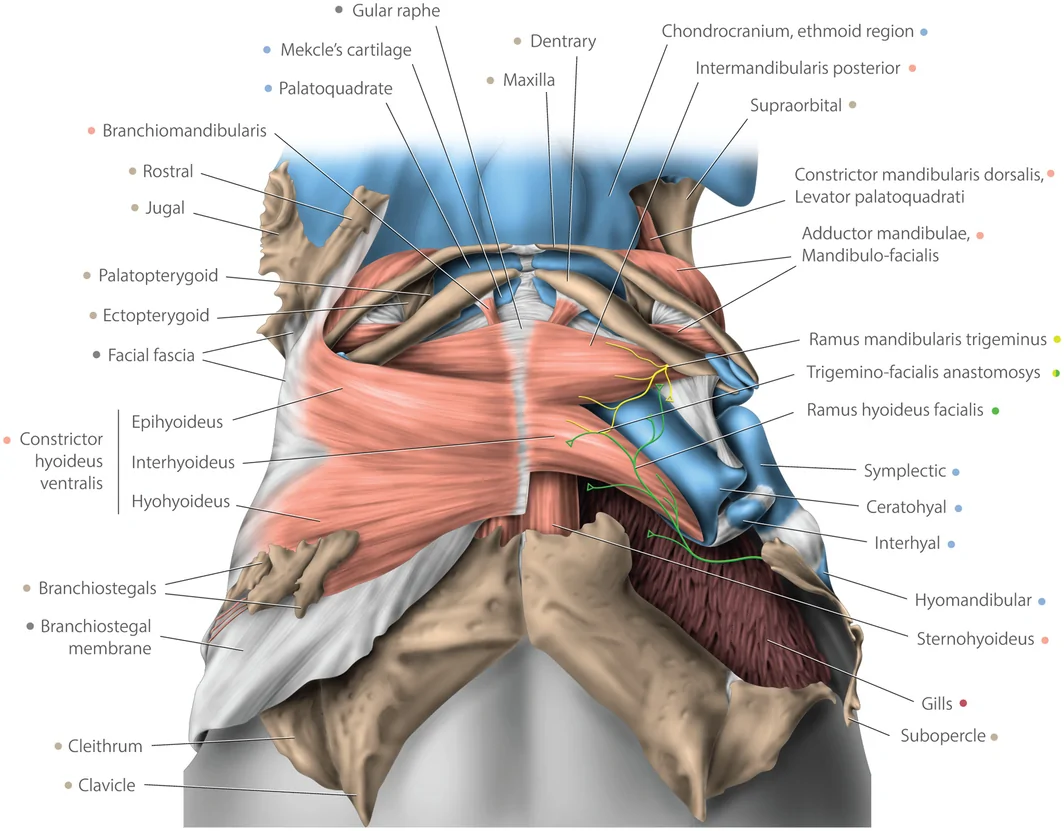
Ventral view of cranial musculoskeletal system and associated major motor branches of trigeminus and facialis nerves of *Acipenser fulvescens* (MZUSP 48364, 121 mm SL). Inverted triangles indicate nerve branches innervating superimposed epihyoideus and hyohyoideus. Right nerves and left jugal, rostrals, branchiostegal membrane, medial branchiostegals, epihyoideus, and hyohyoideus removed.

**FIGURE 4:**
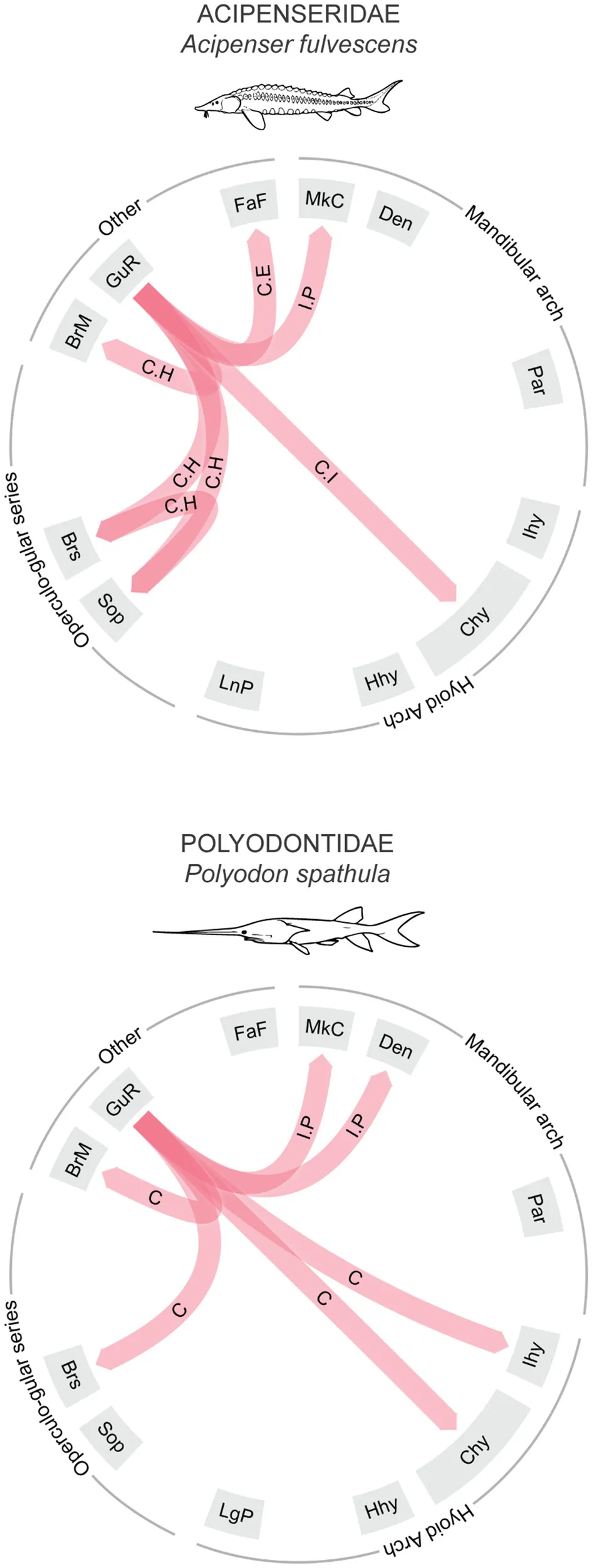
Connection diagrams of gular muscles in representatives of chondrostean fishes. Primarily compressive actions indicated in red. Straight and pointed tips indicate muscle origin and insertion, respectively. BrM, branchiostegal membrane; Brs, branchiostegals; C, constrictor hyoideus ventralis; C.E, epihyoideus; C.H, hyohyoideus; C.I, interhyoideus; Chy, ceratohyal; Cor, coronoid; Den, dentary; FaF, facial subdermal fascia; GuR, gular raphe; Hhy, hypohyal; I, intermandibularis; I.P, intermandibularis posterior; Ihy, interhyal; LnP, lingual pad; MkC, Meckel's cartilage; Par, prearticular; Sop, subopercle.

The intermandibularis anterior is absent. The intermandibularis posterior is attached laterally to Meckel's cartilage and medially to a gular aponeurosis that narrows posteriorly and assumes the shape of a raphe.

The ventral portion of the intermandibularis posterior is partially continuous with a superficial set of fibers that spreads out into a connective, membranous tissue that extends dorsolaterally and covers all muscles and most skeletal elements of the lateral surface of the head. This set of fibers, herein termed the epihyoideus, is also partially continuous ventrally with the gular muscles derived from the hyoid arch, that is, the interhyoideus and hyohyoideus (described below). The homology of epihyoideus is considered in section 4.1.

The interhyoideus is fully covered ventrally by the epihyoideus. The interhyoideus originates from the gular raphe, which is shared anteriorly with the intermandibularis posterior, epihyoideus, and hyohyoideus. Insertion is on the posteromedial region of the ceratohyal.

Fibers of the hyohyoideus arise from the gular raphe shared with the other gular muscles and spread out into the branchiostegal membrane and attach to the branchiostegals and ventral portion of the subopercle.

Except for small branches from the ramus mandibularis trigeminus innervating the intermandibularis posterior, the main trunk of the nerves that serve the gular musculature runs between the outermost muscles epihyoideus and hyohyoideus and the deeper intermandibularis posterior and interhyoideus. Branches that diverge early from the main trunk of the ramus mandibularis trigeminus innervate the intermandibularis posterior and part of the epihyoideus. At the level of the middle of the interhyoideus, a posterior branch of the ramus mandibularis trigeminus anastomoses with a dorsal branch of the ramus hyoideus facialis. Branches that diverge after that anastomosis innervate the epihyoideus and the interhyoideus. Branches of the ramus hyoideus facialis emerging prior to the anastomosis innervate the hyohyoideus, epihyoideus, and interhyoideus.

##### Polyodontidae (Figure 4)

The intermandibularis anterior is absent. Fibers of the intermandibularis posterior arise medially from a gular raphe that extends posteriorly to the constrictor hyoideus ventralis. The muscle attaches laterally to Meckel's cartilage and the dentary.

The constrictor hyoideus ventralis is undivided and partially continuous anteromedially with the intermandibularis. The constrictor hyoideus ventralis arises from the gular raphe and inserts on the posterior half of the ceratohyal, interhyal, and branchiostegal.

Branches of the ramus mandibularis trigeminus innervate the intermandibularis posterior and at least some anterior fibers of the constrictor hyoideus ventralis. Branches of ramus hyoideus facialis innervate the constrictor hyoideus ventralis. We were unable to detect anastomoses between the branches of the ramus hyoideus facialis and the ramus mandibularis trigeminus. However, these nerves are so intensely reticulated at the anteriormost region of the constrictor hyoideus ventralis that we cannot rule out the possibility of some anastomoses occurring between their smallest branches.

#### 3.1.3 Holostei

##### Amiiformes (Figures 5 and 6)

**FIGURE 5:**
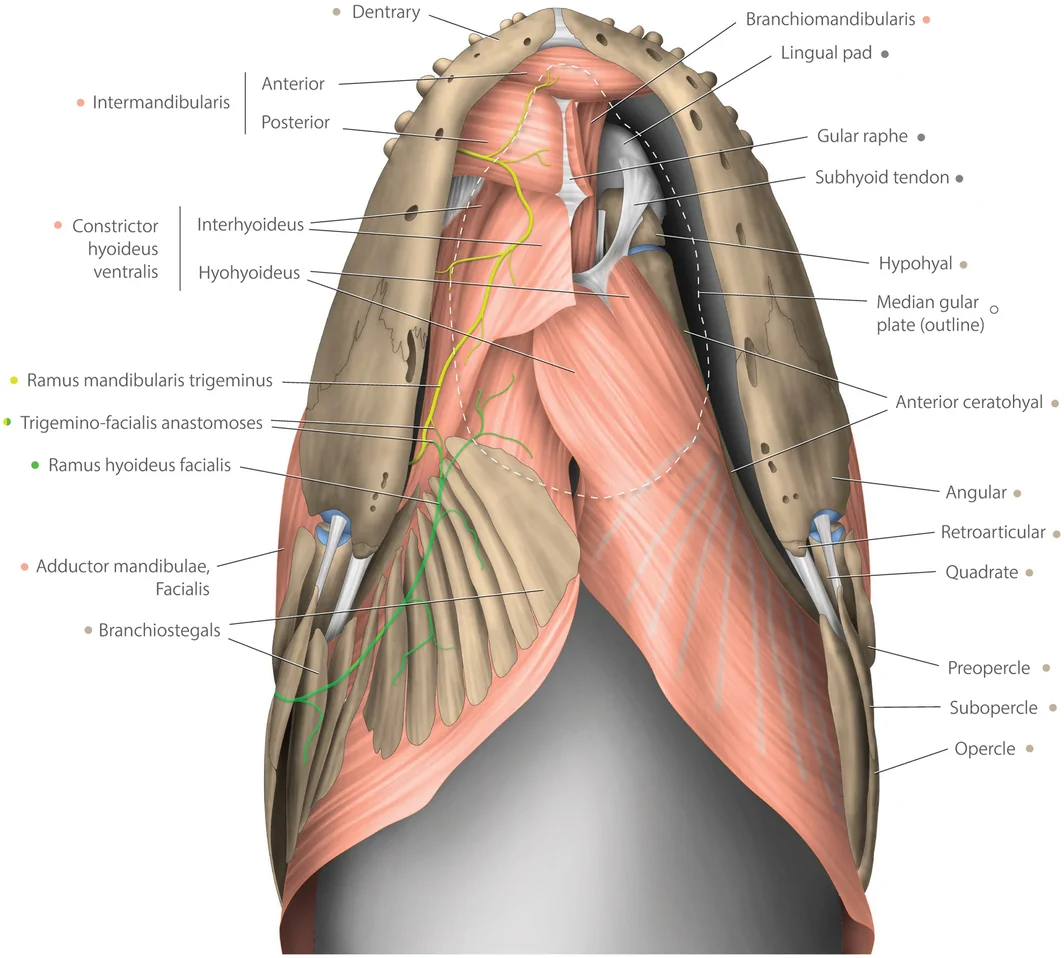
Ventral view of cranial musculoskeletal system and associated major motor branches of trigeminus and facialis nerves of *Amia calva* (USNM 64338, 176 mm SL). Median gular plate and left branchiostegals, intermandibularis posterior, and interhyoideus removed. Contour of median gular plate represented by dashed line.

**FIGURE 6:**
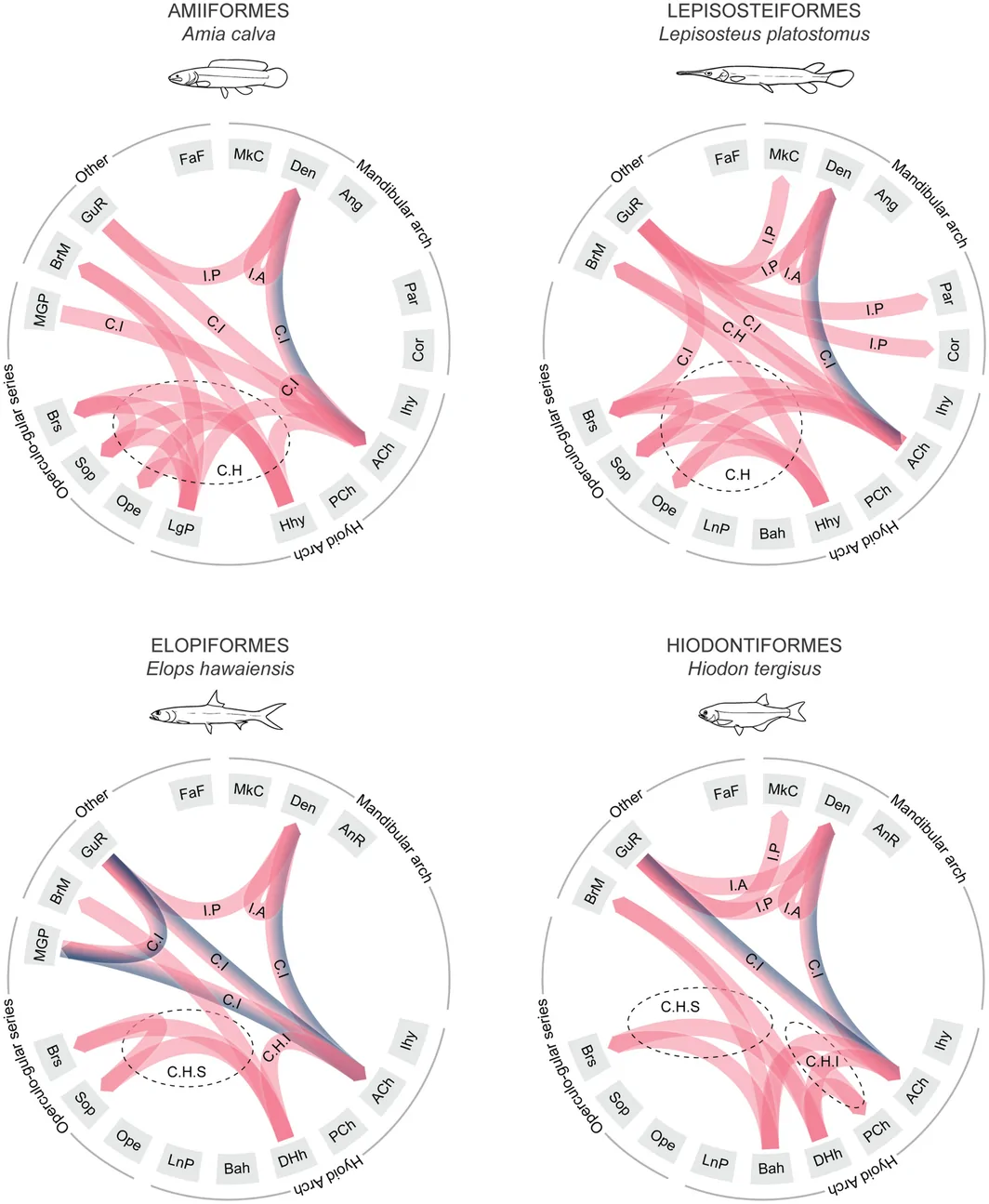
Connection diagrams of gular muscles in representatives of neopterygian fishes. Primarily expansive and compressive actions indicated in blue and red, respectively. Straight and pointed tips indicate muscle origin and insertion, respectively. Ang, angular; AnR, angulo‐retroarticular; Ach, anterior ceratohyal; Bah, basihyal; BrM, branchiostegal membrane; Brs, branchiostegals; C, constrictor hyoideus ventralis; C.H, hyohyoideus; C.H.I, hyohyoideus inferior; C.H.S, hyohyoideus superior; C.I, interhyoideus; Cor, coronoid; Den, dentary; DHh, dorsal hypohyal; FaF, facial subdermal fascia; GuR, gular raphe; Hhy, hypohyal; I, intermandibularis; I.A, intermandibularis anterior; I.P, intermandibularis posterior; Ihy, interhyal; LnP, lingual pad; MGP, mediam gular plate; MkC, Meckel's cartilage; Ope, opercle; Par, prearticular; PCh, posterior ceratohyal; Sop, subopercle.

The intermandibularis is divided into an anterior and a posterior section. Fibers of the intermandibularis anterior are uninterrupted along the entirety of the muscle and attach to the anteroventral region of the dentaries. The gular raphe serves as the main site of origin of the intermandibularis posterior. A few anterodorsal fibers of this section originate from an aponeurosis for the origin of the ventromedial section of the interhyoideus. Toward insertion, the intermandibularis posterior becomes aponeurotic and attaches to a sulcus on the dentary located just ventral to Meckel's cartilage.

Anteriorly, the interhyoideus is completely separated into a medial and a lateral section. The former contacts its antimere as it arises from the gular raphe shared with the posterior section of the intermandibularis. This region of the gular raphe attaches to the median gular plate, thus indirectly connecting this plate with the interhyoideus medial. Posterior to that point, the fibers of the section are fully continuous along the contralateral halves of the muscle. The interhyoideus lateral arises from flattened tendons deeply embedded into the connective membrane forming the floor of the buccal cavity, just dorsal to the intermandibularis. The interhyoideus lateral has no contact with its antimere. Near the posterior insertion, the two sections of the interhyoideus merge with each other, and the entire muscle attaches to the lateral region of the anterior ceratohyal. The posteromedial border of the muscle passes dorsal to the bases of the branchiostegals but usually does not attach to these bones; the only observed exception was an attachment of a few fibers to the base of the fifth medialmost branchiostegal of the right side.

The hyohyoideus is partially differentiated at origin into lateral and medial sections. Both sections originate from a V‐shaped subhyoid tendon that anchors to the ventrolateral regions of the hypohyal and lingual pad (strong connective tissue forming the tip of the tongue). In the region of origin, the left hyohyoideus overlaps ventrally with its antimere. Toward insertion, lateral and medial sections are no longer distinguishable and the entire hyohyoideus inserts on the anterior ceratohyal, branchiostegal membrane, branchiostegals, subopercle, and opercle.

Branches diverging earlier from the main trunk of the ramus mandibularis trigeminus innervate the intermandibularis anterior, intermandibularis posterior, and at least the anterior half of the interhyoideus. Distal branches of the ramus mandibularis trigeminus then anastomose with apical branches of the ramus hyoideus facialis at the posterior portion of the interhyoideus. Prior to those anastomoses, branches of the ramus hyoideus facialis innervate the hyohyoideus.

##### Lepisosteiformes (Figure 6)

The intermandibularis covers most of the ventral surface of the head. The muscle is undivided, but two portions are easily distinguishable: a short anterior section, with fibers uninterrupted along the entire expanse of the muscle and attaching only to the contralateral dentaries, and an extensive posterior section in which the fibers associate primarily with a gular raphe. The posterior section attaches primarily to the dentary, coronoid (=coronoid 2), Meckel's cartilage, and prearticular. A few posterolateral fibers of this section converge medially to a tendinous tissue that connects with some superficial fibers of the interhyoideus.

The interhyoideus is discreetly separated anteriorly into a medial and a lateral section. The former contacts its antimere at origin via a gular aponeurosis formed as a posterior expansion of the gular raphe of the intermandibularis posterior. The larger lateral section arises from a broad connective sheet extending through most of the floor of the lower jaw, just dorsal to the intermandibularis. The interhyoideus lateral has no connection with its antimere. Shortly after their separate origins, both sections of the interhyoideus merge with one another and the entire muscle inserts on the posterior region of the anterior ceratohyal and the head of the medialmost branchiostegal. Near its insertion, the posteromedial region of the interhyoideus is continuous with the hyohyoideus.

The hyohyoideus emerges anteromedially from a V‐shaped subhyoid tendon that anchors to the ventral hypohyal and anterior ceratohyal. From its origin, the muscle spreads through the connective tissue of the branchiostegal membrane and attaches to the branchiostegals, subopercle, and opercle.

The innervation pattern of the gular muscles is primarily similar to that observed in *Amia*.

#### 3.1.4 Teleostei

##### Elopiformes (Figures 6 and 7)

**FIGURE 7:**
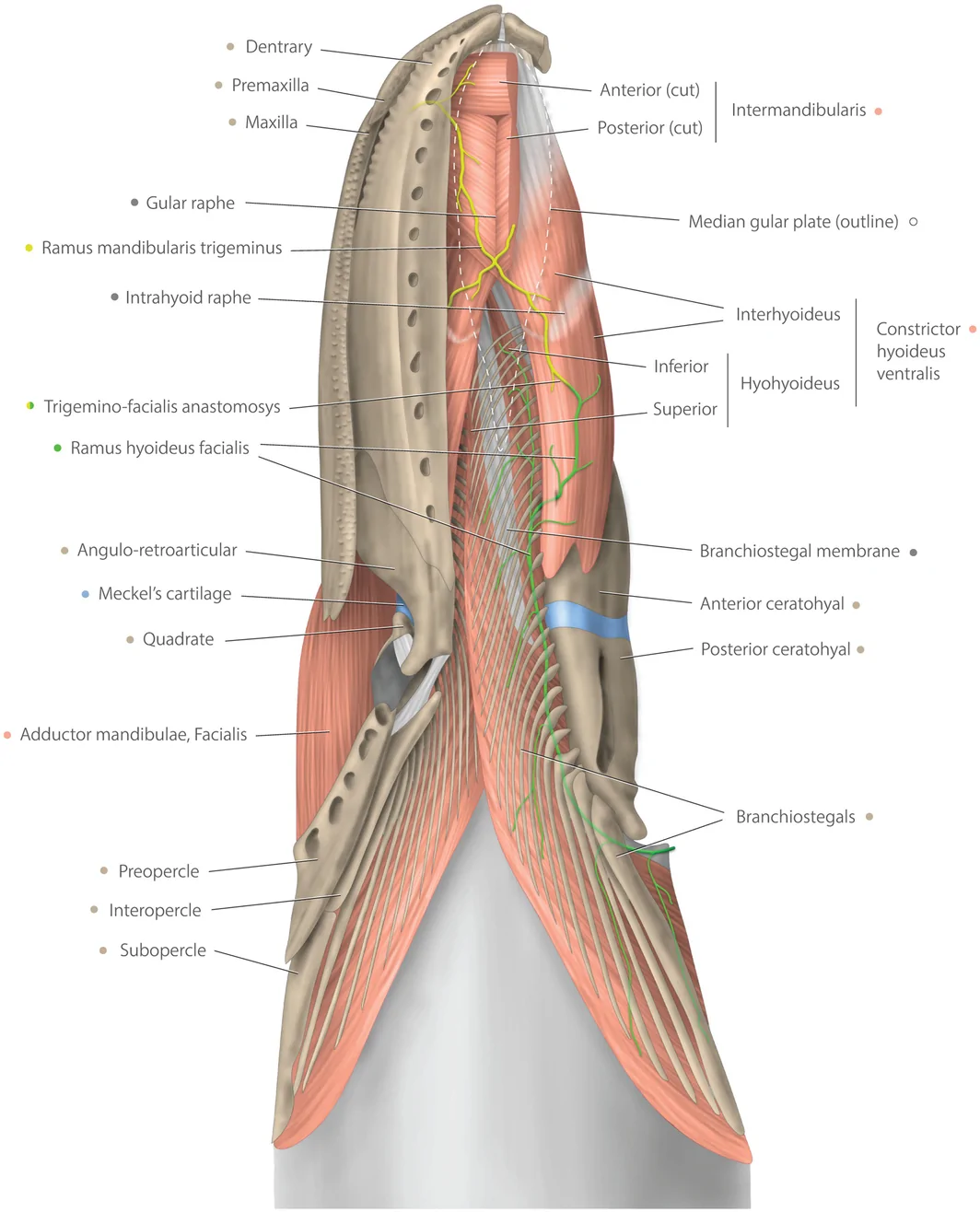
Ventral view of cranial musculoskeletal system and associated major motor branches of trigeminus and facialis nerves of *Elops lacerta* (MZUSP 84787, 111 mm SL). Median gular plate, right facialis nerve, and left jaws, suspensorium, opercular series, facial muscles, and anterior portion of ramus mandibularis trigeminus removed. Contour of median gular plate represented by dashed line.

The intermandibularis has both aponeurotic and musculous attachments to the anteroventral region of the medial face of the dentary. Medially, the muscle separates into anterior and posterior subdivisions. Fibers of the former are uninterrupted along the entire extent of the muscle between the dentaries. The intermandibularis posterior converges posteriorly to the gular raphe shared with part of the interhyoideus.

The interhyoideus arises primarily from an aponeurosis attached to the symphyseal region of the dentaries. This aponeurosis passes dorsal to the intermandibularis and is partially embedded into the connective tissues forming the roof of the buccal cavity. Posteriorly, the aponeurosis for the origin of the interhyoideus narrows to form a gular raphe that serves as the origin site for the medial fibers of the muscle. This raphe is fully continuous ventrally with that associated with the intermandibularis posterior. The interhyoideus exhibits a conspicuous transverse aponeurosis nearly at its mid‐length, thus superficially partitioning the muscle into an anterior and a posterior segment. We designate this raphe as the intrahyoid raphe. The muscle proceeds posteriorly until it attaches to the posterior region of the anterior ceratohyal. Two sets of fibers of the interhyoideus discreetly attach to the median gular plate: the lateralmost fibers of the posterior segment converge to tendons anchoring to the anterolateral border of the plate, and a few medialmost fibers of the anterior segment of the muscle directly attach to the dorsoposterior region of the plate.

The origin of the hyohyoideus is from a V‐shaped subhyoid tendon anchored to the posterolateral region of the dorsal hypohyal. Medially, the left hyohyoideus overlaps ventrally with its antimere. The muscle proceeds posteriorly, passing medial to the branchiostegals and completely subdividing into two muscles toward insertion. The hyohyoideus inferior inserts on the posteroventral border of the anterior ceratohyal, whereas the hyohyoideus superior radiates through the branchiostegal membrane, attaching to the medial sides of the branchiostegals and subopercle.

Branches of the ramus mandibularis trigeminus innervate the entire intermandibularis. The contralateral main trunks of this nerve meet each other sagittally, forming a chiasma at the level of the ventral connection between the intermandibularis posterior and interhyoideus. The main branch of the ramus mandibularis trigeminus, posterior to that chiasma, proceeds posteroventrally over the interhyoideus, giving off small branches that innervate the anterior portion of the muscle. At the mid‐length of the posterior segment of the interhyoideus, the main trunks of the ramus mandibularis trigeminus and ramus hyoideus facialis form an anastomosis. Branches of the latter nerve innervate at least the posterior portion of the interhyoideus and the whole hyohyoideus.

##### Hiodontiformes (Figure 6)

The intermandibularis exhibits a complex architecture with several subdivisions. The intermandibularis anterior attaches to the anteroventral region of the medial face of the dentaries. Most fibers of this muscle are uninterrupted between the contralateral dentaries, but the ventromedial fibers attach medially to a broad gular aponeurosis present along the ventral face of the head musculature. The intermandibularis posterior is subdivided into three sections. The dorsal section emerges from the anterior regions of the dentary and Meckel's cartilage and converges ventromedially to a tendon for the origin of the lateral portion of the interhyoideus (see below). The middle section extends between this same insertional tendon and the gular aponeurosis. Fibers of the ventral section of the intermandibularis posterior run from this aponeurosis to the anteroventral portion of the dentary.

The interhyoideus attaches to the posterior region of the anterior ceratohyal, including its cartilaginous end. Anterolaterally, the muscle exhibits a broad, ventral intrahyoid aponeurosis that associates with the superficial but not the deeper fibers of the muscle. As the muscle proceeds anteriorly, most fibers converge to the gular aponeurosis shared with the intermandibularis posterior. The lateralmost fibers of the interhyoideus, in turn, converge to an elongate tendon that passes dorsal to the intermandibularis until attaching to the symphyseal region of the dentary. This tendon is embedded in the connective tissue forming the floor of the buccal cavity and also receives fibers of the dorsal and middle section of the intermandibularis posterior (see above).

The hyohyoideus originates from a W‐shaped subhyoid tendon that anchors medially to the basihyal cartilage and laterally to the dorsal hypohyal. Toward insertion, the muscle passes dorsal to the first branchiostegals, and after the second branchiostegal, it splits into the inferior and superior partes. The hyohyoideus inferior does not attach to any branchiostegals and inserts along the cartilaginous ventral borders of the anterior and posterior ceratohyals. The hyohyoideus superior attaches to the branchiostegals and branchiostegal membrane.

The innervation pattern of the gular muscles is mostly similar to that of *Elops*.

### 3.2 Phylogenetic inferences

To phylogenetically interpret the early transformations in the actinopterygian gular musculature, we compared the conditions observed in the described basal actinopterygians with those of more derived actinopterygian lineages (16 additional orders of pre‐acanthomorph teleosts), as well as key representatives of sarcopterygian, cartilaginous, and jawless fishes (Table 1). The observed transformations were organized into the following phylogenetic characters and subsequently mapped onto a time‐calibrated phylogeny of the examined gnathostome fishes (Figure 8) using stochastic ancestral state reconstructions (Figures 9 and 10).

**FIGURE 8:**
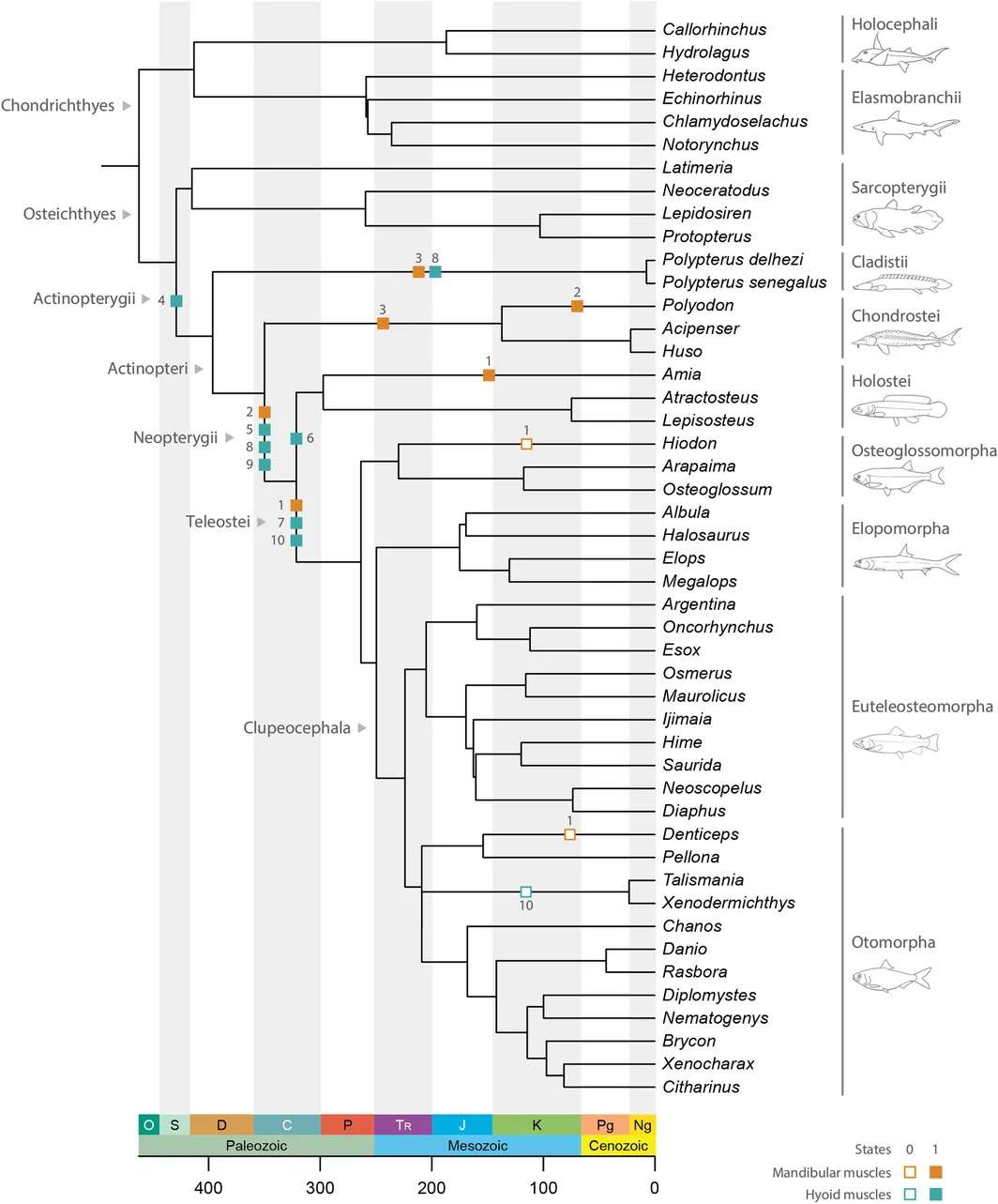
Evolutionary transformations of gular muscles mapped onto a time‐calibrated phylogenetic tree of examined gnathostome fishes. Tree extracted from Kumar et al. (2022). Synapomorphies based on stochastic ancestral state reconstructions (Figures 9 and 10); character definitions provided in section 3.2.

**FIGURE 9:**
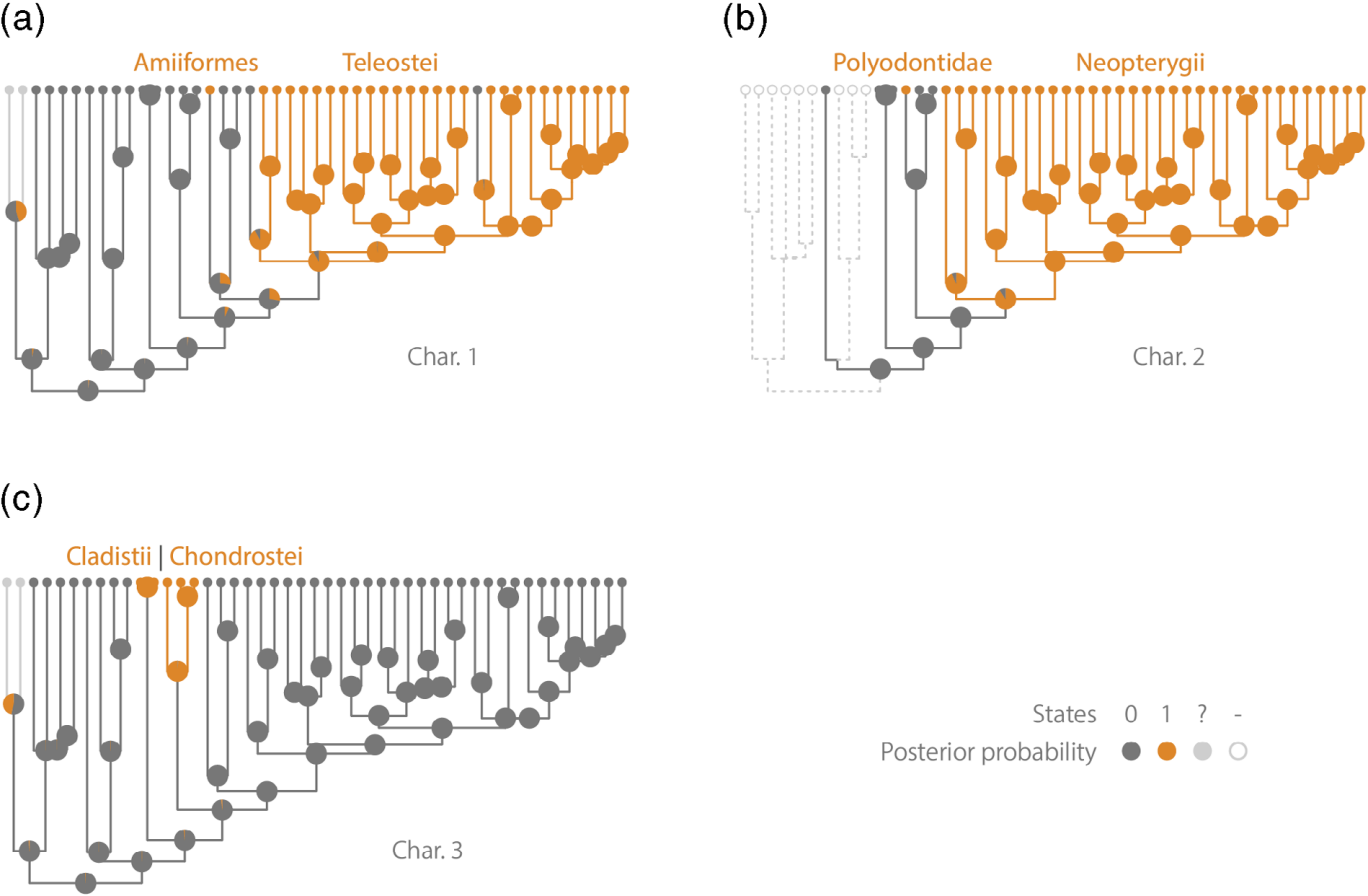
Stochastic ancestral state reconstructions of mandibular gular muscle characters (Chars. 1–3), with posterior probabilities represented as pie charts. See Figure 8 and section 3.2 for details on tree topology and character definitions.

**FIGURE 10:**
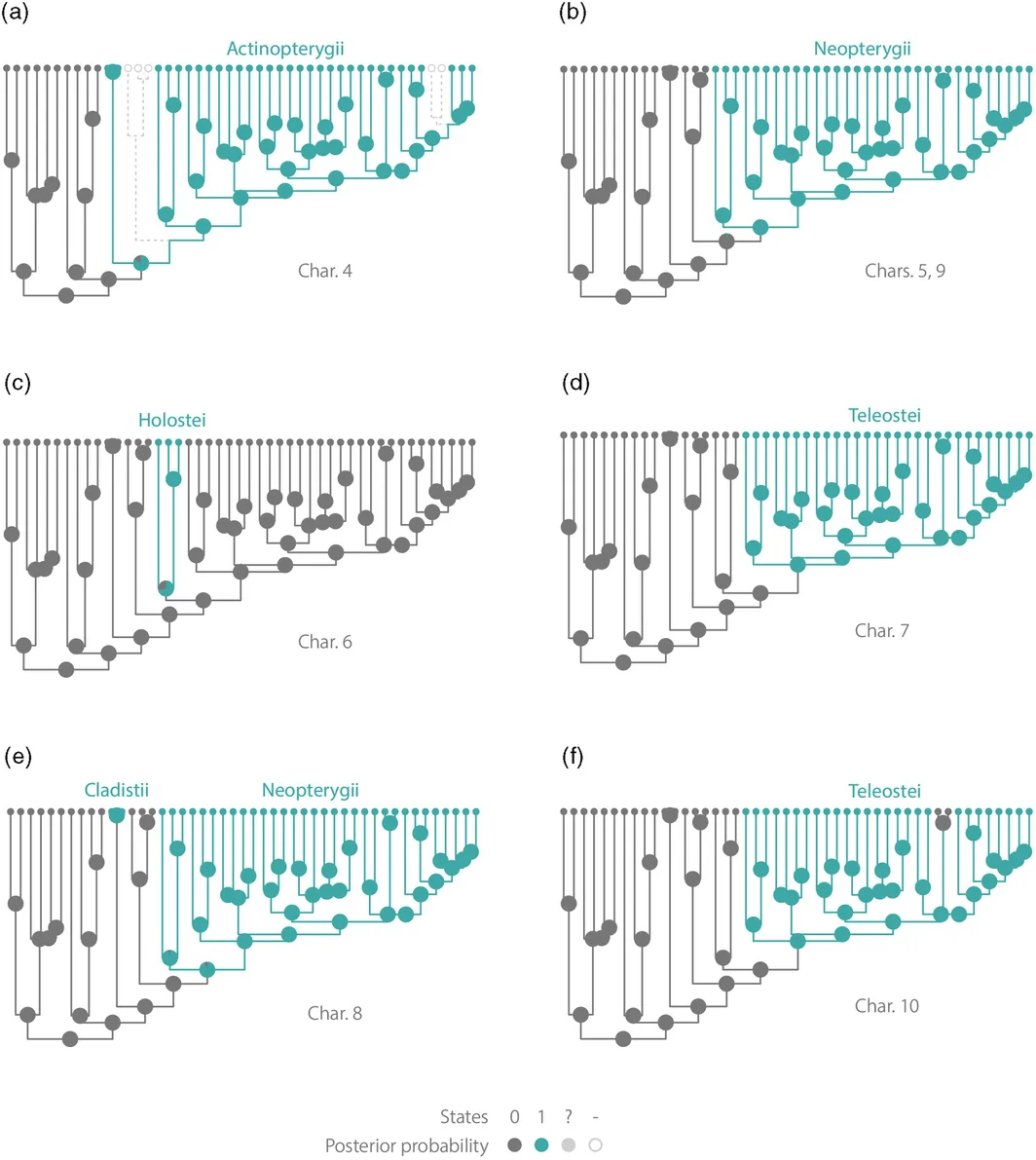
Stochastic ancestral state reconstructions of hyoid gular muscle characters (Chars. 4–10), with posterior probabilities represented as pie charts. See Figure 8 and section 3.2 for details on tree topology and character definitions.

#### 3.2.1 1. Intermandibularis, insertion on Meckel's cartilage: (0) present; (1) absent; (?) indeterminate

Meckel's cartilage is a plesiomorphic insertion site for the intermandibularis in jawed fishes. This condition was observed in the examined elasmobranchs, sarcopterygian fishes, polypteriforms, acipenseriforms, lepisosteiforms, hiodontiforms, and the clupeiform *Denticeps* (Figures 2, 3, 4, 6, and 11). In all other examined bony fishes, this attachment is absent, and the intermandibularis inserts instead onto other skeletal elements of the lower jaw (Figure 6).

**FIGURE 11:**
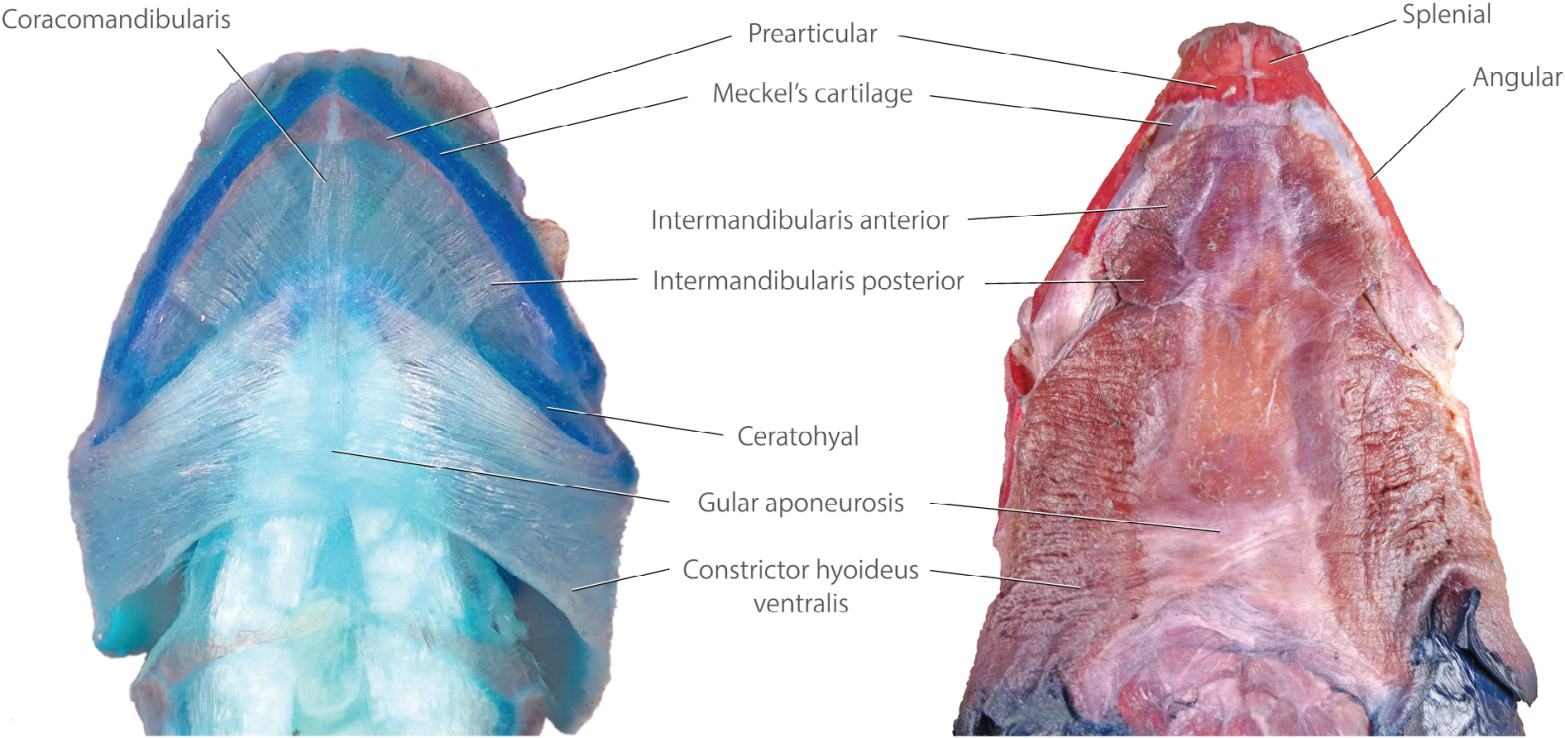
Ventral view of cranial musculoskeletal system of *Neoceratodus forsteri* in (a) larval (USNM 327317, 11 mm TL) and (b) adult (USNM 40011, 502 mm TL) stages. Not to scale.

Although some studies have identified certain holocephalans muscles as intermandibularis (Didier, 1995; Edgeworth, 1935), other works propose that these are actually pre‐mandibular muscles, homologous to the labial muscles of agnathans (Kesteven, 1933; Luther, 1909a; Vetter, 1878). Evidence supporting the latter interpretation includes the observation that these muscles are never attached to the lower jaw, but are exclusively attached to the labial cartilages. Due to these uncertainties, the condition in Holocephali was coded as indeterminate (?).

Stochastic ancestral state reconstructions (best‐fitting model = ER; Data S2) indicate that the attachment of the intermandibularis to Meckel's cartilage was likely lost independently in amiiforms and teleosts (posterior probability, PP = 91.7%; Figures 8 and 9). Independent reversal to the ancestral state within Teleostei would have occurred in *Hiodon* and *Denticeps*.

#### 3.2.2 2. Intermandibularis, insertion on the dentary: (0) absent; (1) present; (‐) inapplicable

The dentary is one of the insertion sites for the intermandibularis in polyodontids and neopterygians (Figures 5, 6, 7). The muscle never attaches to the dentary in actinistians, polypteriforms, or acipenserids (Figures 2 and 4). This character is inapplicable for chondrichthyans and dipnoans because they lack a dentary (Figure 11).

Stochastic ancestral state reconstructions (best‐fitting model = ARD; Data S2) identify the attachment of the intermandibularis onto the dentary as independent synapomorphies for Polyodontidae (PP = 100%) and Neopterygii (PP = 90.8%; Figures 8 and 9).

#### 3.2.3 3. Intermandibularis anterior: (0) present; (1) absent; (?) indeterminate

In most taxa examined, the intermandibularis extends from the mandibular symphysis anteriorly to the region of contact with the interhyoideus posteriorly in adults (Figures 5, 7, and 11). In bony fishes, this muscle often differentiates into an intermandibularis anterior, located near the symphysis, and an intermandibularis posterior, associated with the gular raphe. In polypteriforms (Cladistii) and acipenseriforms (Chondrostei), the intermandibularis is restricted to the posterior portion associated with the gular raphe (Figures 1 and 3). No muscular fibers occur near the mandibular symphysis, and as a result, a considerable portion of the branchiomandibularis (=coracomandibularis) is exposed in ventral view. This condition suggests that polypteriforms and acipenseriforms possess only the intermandibularis posterior, whereas the anterior intermandibularis is absent.

The examined hexanchiforms, lepidosirenids, and protopterids do not exhibit two differentiated intermandibulares muscles in adults. However, unlike the condition in polypteriforms and acipenseriforms, this undivided muscle extends anteriorly up to the symphyseal region of the lower jaw. This leads us to interpret that no portion of the intermandibularis has been lost in hexanchiforms, lepidosirenids, and protopterids, and that the muscle is simply undifferentiated into anterior and posterior sections. The same condition appears to occur in other groups of cartilaginous fishes (Luther, 1909b). Because the lack of differentiation of the intermandibularis is restricted to some non‐actinopterygian groups, this condition was not coded in the present analysis. Holocephalans were coded as indeterminate (?) due to uncertainties regarding the identity of the so‐called intermandibularis in the group (see Char. 1).

Stochastic ancestral state reconstructions (best‐fitting model = ER; Data S2) recover independent losses of the intermandibularis anterior in Cladistii (PP = 100%) and Chondrostei (PP = 98.6%; Figures 8 and 9).

#### 3.2.4 4. Constrictor hyoideus, dorsoventral continuity: (0) uninterrupted; (1) interrupted between opercle and subopercle; (‐) inapplicable

In lampreys and elasmobranchs, the constrictor hyoideus is a continuous muscle that runs along the entire membrane anterior to the first branchial slit (Figure 2). Sarcopterygian fishes also have this muscle mostly continuous, including fibers running between the opercular and subopercular cartilages and/or ossifications (Figure 9). In actinopterygians, there are no constrictor hyoideus fibers between the opercle and the subopercle (Figures 2, 4, and 6). This condition is thus reconstructed as a synapomorphy for Actinopterygii (PP = 82.1%; best‐fitting model = ARD; Data S2 and Figures 8 and 10). Acipenseriforms and siluriforms lack the opercle and subopercle, respectively, and are therefore inapplicable for this character.

#### 3.2.5 5. Constrictor hyoideus ventralis, interhyoideus, direct attachment to the lower jaw: (0) absent; (1) present

Primitively in gnathostomes, the anterior portion of the constrictor hyoideus ventralis, i.e., the interhyoideus, runs from the gular raphe to the posterior region of the hyoid bar (Figures 1, 2, 3, 4 and 11). In neopterygians, the interhyoideus is anterolaterally expanded so that its lateral fibers run lateral to the hyoid bar and tendinously attach directly to the lower jaw (Figures 5, 6, 7). Stochastic ancestral state reconstructions (best‐fitting model = ER; Data S2) identify the attachment of the intermandibularis onto the dentary as an evolutionary novelty of Neopterygii (PP = 98.9%; Figures 8 and 10).

#### 3.2.6 6. Constrictor hyoideus ventralis, interhyoideus, partial subdivision into lateral and medial sections: (0) absent; (1) present

Holosts have the interhyoideus partially separated into medial and lateral sections (Figure 5). A comparable subdivision is absent in all other examined gnathostomes (Figures 1, 3, 7, 11, and 12). Stochastic ancestral state reconstructions (best‐fitting model = ER; Data S2) recover the subdivided interhyoideus as an evolutionary novelty of Holostei (PP = 71.8%; Figures 8 and 10).

**FIGURE 12:**
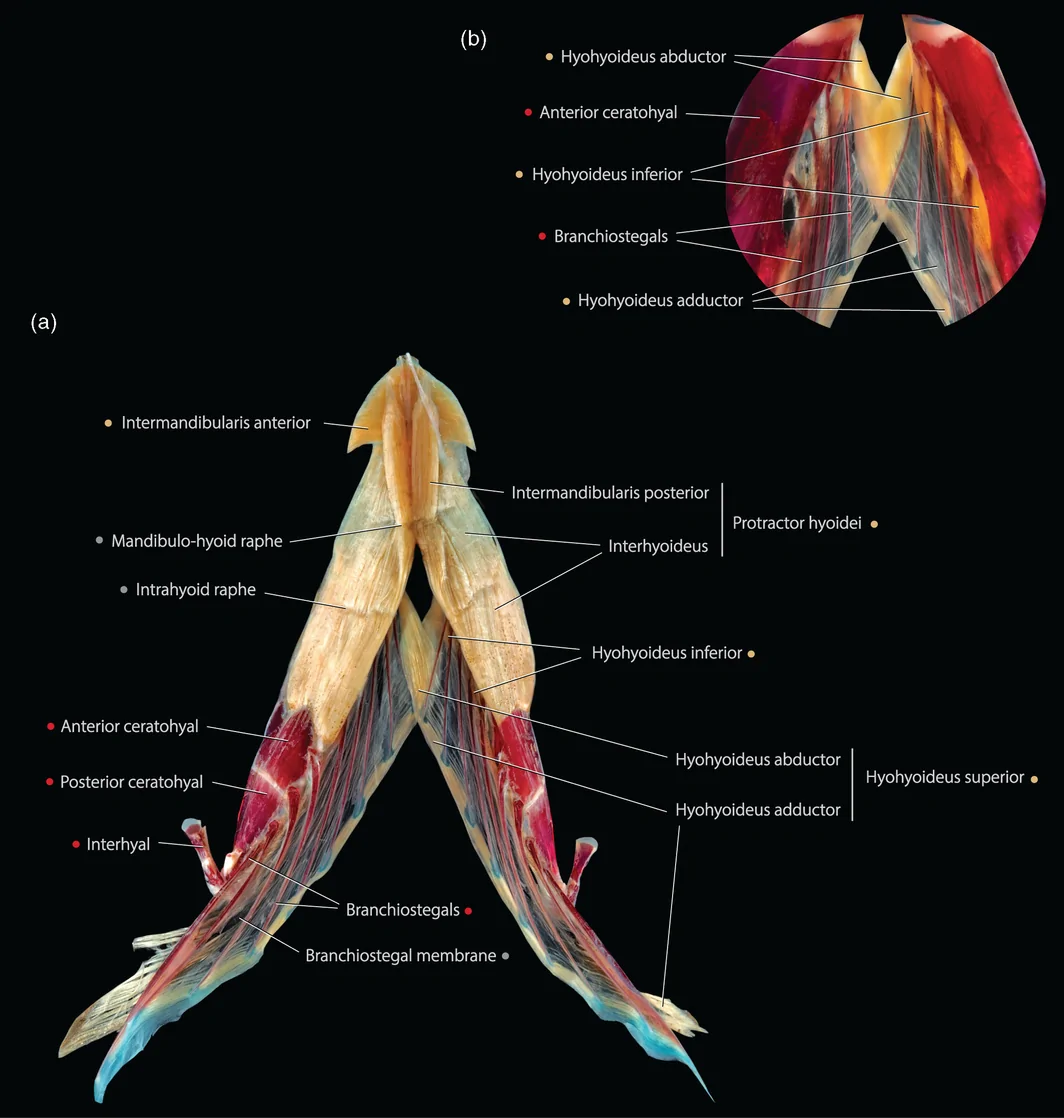
Gular muscles and associated hyoid bar and branchiostegals of *Neoscopelus macrolepidotus* (USNM 301041, 128.6 mm SL). (a) Ventral view; (b) dorsal view of central area showing details of hyohyoidei subdivisions.

#### 3.2.7 7. Constrictor hyoideus ventralis, interhyoideus, intrahyoid raphe or aponeurosis on the ventral surface: (0) absent; (1) present

Teleosts have a short intermediate aponeurosis or raphe on the ventral face of the interhyoideus (Figures 7 and 12). We have named this structure the intrahyoid raphe or aponeurosis. In small specimens, this tendinous formation is entirely transparent, making it challenging to observe at first glance. Nevertheless, we could unequivocally identify it in all teleosts we examined. The intrahyoid raphe should not be confused with the mandibulo‐hyoid raphe. This more anterior transverse raphe marks the ontogenetic fusion of the intermandibularis posterior and the interhyoideus to form the so‐called protractor hyoidei (Figure 12; see section 4.1).

Stochastic ancestral state reconstructions (best‐fitting model = ER; Data S2) identify the presence of the intrahyoid raphe as a synapomorphy for Teleostei (PP = 99.8%; Figures 8 and 10).

#### 3.2.8 8. Constrictor hyoideus ventralis, hyohyoideus, primary origin from the: (0) gular raphe; (1) subhyoid tendon

Primitively in gnathostomes, the entire constrictor hyoideus ventralis originates from the gular raphe, a raphe at the sagittal line that often serves as an origin site for many gular muscles (Figures 2 and 11). In polypteriforms and neopterygians, the posterior subdivision of the muscle, that is, the hyohyoideus, originates entirely or primarily from a V‐ or W‐shaped tendinous formation that lies deeper (dorsal) to the gular raphe (Figures 1, 5, and 7). This tendinous formation is referred to here as the subhyoid tendon. This tendon and its association with the hyohyoideus are not found elsewhere in jawed fishes. Stochastic ancestral state reconstructions (best‐fitting model = ARD; Data S2) indicate that the subhyoid tendon evolved independently in Cladistii (PP = 100%) and Neopterygii (PP = 94.3%; Figures 8 and 10).

#### 3.2.9 9. Constrictor hyoideus ventralis, hyohyoideus, origin from the anterior portion of the hyoid bar: (0) absent; (1) present

In cartilaginous and lobe‐finned fishes, the posterior portion of the constrictor hyoideus ventralis, that is, the hyohyoideus, originates only from the gular raphe (Figures 2 and 11). In polypteriforms, the hyohyoideus arises from both this raphe and the underlying subhyoid tendon (Figure 1). Although this tendon proceeds dorsally to the gular raphe, it is anchored to the walls of the sternohyoideus and never reaches the hyoid bar. The subhyoid tendon in neopterygians advances more dorsally until it attaches to an ossification on the anterior portion of the hyoid bar (hypohyal, dorsal hypohyal, ventral hypohyal, or anterior ceratohyal; Figures 5 and 6). Stochastic ancestral state reconstructions (best‐fitting model = ER; Data S2) recover this condition as an evolutionary novelty in Neopterygii (PP = 98.9%; Figures 8 and 10).

#### 3.2.10 10. Constrictor hyoideus ventralis, hyohyoideus, distal separation into inferior and superior subdivisions: (0) absent; (1) present

In most examined teleosts, the hyohyoideus is laterally subdivided into an anterior hyohyoideus inferior and a posterior hyohyoideus superior (Figures 6, 7, and 12). The former muscle is primarily associated with the posterior end of the hyoid bar and the bases of the branchiostegals, whereas the latter is associated with the branchiostegal membrane and the distal portions of the branchiostegals and opercular series. Other jawed fishes lack this kind of lateral subdivision in the hyohyoideus (Figures 1, 2, 3, 4, 5, 6 and 11). Among the examined teleosts, only alepocephaliforms possess an undivided hyohyoideus. The clupeiform *Pellona*, the stomiiform *Maurolicus*, and the myctophiform *Diaphus* each have a single hyohyoideus muscle, but its bony associations indicate that it corresponds to the hyohyoideus superior only, with the hyohyoideus inferior apparently lost.

Stochastic ancestral state reconstructions (best‐fitting model = ER; Data S2) identify the lateral separation of the hyohyoideus into inferior and superior subdivisions as a synapomorphy of Teleostei (PP = 99.7%) with a subsequent reversal in Alepocephaliformes (PP = 100%; Figures 8 and 10).

## 4 DISCUSSION

### 4.1 Homologies and ontogeny of gular muscles

Our analysis shows that, except for Polypteriformes and Acipenseriformes (Figures 1 and 3), adults of all other examined Actinopterygii possess two subdivisions of the intermandibularis muscle—the anterior and the posterior (Character 3; Figures 2 and 5, 6, 7). Interestingly, the intermandibularis anterior develops late during ontogeny. *Neoceratodus* larvae have only the intermandibularis posterior, while adults have both muscles (Figure 11). The same ontogenetic pattern has been reported for several actinopterygians (Edgeworth, 1929; Schilling & Kimmel, 1997; Uji et al., 2010), suggesting that this is a general pattern of bony fishes at least.

The constrictor hyoideus ventralis, which is primarily an undivided muscle in lampreys and elasmobranchs (Figure 2), has differentiated into several independent subdivisions through osteichthyan evolution. The first and most common of its subdivisions is into an anterior interhyoideus and a posterior hyohyoideus (Figures 1, 2, 3, 4, 5, 6). The former attaches primarily to the hyoid bar and median gular plate, while the latter attaches to the branchiostegal membrane and its associated skeletal elements (lateral gular plates, branchiostegals, and subopercular and opercular bones and/or cartilages). In coelacanths, protopterids, and lepidosirenids, the two muscles are completely continuous medially, but partially separated laterally. Uniquely in non‐acipenseriform actinopterygians, the differentiation between interhyoideus and hyohyoideus is also observed medially by the association of the latter muscle with the subhyoid tendon (Figure 5; see Characters 8 and 9).

In most examined teleosts, the intermandibularis posterior has a more anteroposterior orientation and is more intimately associated with the interhyoideus than the intermandibularis anterior (Figure 12). In most extreme conditions, the intermandibularis posterior and interhyoideus are largely continuous with one another, forming a single functional unity designated as the protractor hyoidei by Greenwood (1971) and Winterbottom (1974) [it should be noted, however, that this usage of the term differs from that of Holmqvist, 1910, 1911 and Luther, 1913; see Data S3]. In such instances, a transversal raphe marks the ontogenetic fusion between the intermandibularis posterior and interhyoideus. We named this raphe the mandibulo‐hyoid raphe. It lies anterior to a second raphe, the intrahyoid raphe, which appears at the ventral surface of the interhyoideus in teleosts (Figures 7 and 12; Character 7). Visualization of the mandibulo‐hyoid and intrahyoid raphes may be difficult in small specimens because of their transparency; however, we unequivocally identified them in all teleosts we examined. Their presence is also relatively well documented in the literature (e.g., Osse, 1969; Winterbottom, 1974).

Studies of muscle ontogeny show that the hyohyoideus grows dorsoventrally in polypteriforms and acipenseriforms (Noda et al., 2017; Warth et al., 2018). The opposite pattern has been reported for distantly related groups of neopterygians (Konstantinidis et al., 2015; Schilling & Kimmel, 1997; Uji et al., 2010, 2014). This suggests that the second pattern may be synapomorphic for Neopterygii. However, further studies on the ontogeny of other actinopterygian taxa are needed to confirm this hypothesis.

In teleosts, the hyohyoideus muscle is further subdivided into hyohyoideus inferior and hyohyoideus superior (Figure 7; Character 10). This pattern is commonly observed during ontogeny, where the initially undivided hyohyoideus later separates into distinct components (Schilling & Kimmel, 1997; Uji et al., 2010, 2014). In some teleosts, the medial and lateral portions of the hyohyoideus can become more developed and partially distinct from the remainder of the muscle. In such cases, the medialmost portion, which extends between the origin at the dorsal hypohyal and the first or second branchiostegal, is sometimes referred to as the hyohyoideus abductor (Figure 12) (Winterbottom, 1974). The remainder of the muscle is then termed the hyohyoideus adductor (Figure 12) (Winterbottom, 1974). This differentiation apparently arose multiple times during teleost radiation, and alternative patterns of subdivision of the hyohyoideus superior may have led to different sections being named hyohyoideus abductor and hyohyoideus adductor (cf., siluriforms and gobiiforms). Moreover, in many neoteleosts, the hyohyoideus inferior attaches to the proximal portions of the branchiostegals (Figure 10), and some authors have also referred to this muscle portion as the hyohyoideus abductor due to its presumed functional role (Winterbottom, 1974). As a result, the use of the terms hyohyoideus abductor and hyohyoideus adductor is ambiguous and may not reflect true homology across disparate teleost groups. A comprehensive mapping of hyohyoideus superior differentiation would require broader taxonomic sampling and falls beyond the scope of the present study. In most of the teleosts examined here, differentiation into abductor and adductor components is unclear or absent (e.g., De Schepper et al., 2007); thus the designation hyohyoideus superior is more appropriate in these cases.

The epihyoideus is a muscle unique to acipenserids (Figures 3 and 4). In adults, this muscle is partially continuous with both the intermandibularis posterior and the interhyoideus. Warth et al. (2018) interpreted this muscle as being an “intermandibularis posterior” (and our intermandibularis posterior as “intermandibularis anterior”). However, during its initial appearance in development, the muscle is partially continuous with the constrictor hyoideus ventralis (Warth et al., 2018, p. 168), and it only fuses with the intermandibularis posterior in the latter stages. These indicate that the muscle is a hyoid, not a mandibular muscle. Moreover, comparisons with other bony fishes indicate that the intermandibularis muscle in acipenserids is the posterior one, as it does not reach the mandibular symphysis (see Character 3).

### 4.2 Innervation patterns

The association between cranial muscles and their innervating cranial nerves has long been considered invariant across vertebrates by many studies. This paradigm, known as nerve‐muscle specificity, originates from early comparative studies published in the late 19th century (Cunningham, 1881; Fürbringer, 1888; Gegenbaur, 1874) and has been perpetuated by numerous authors who have relied on patterns of innervation to name and homologize cranial muscles. Despite its broad acceptance among some morphologists, many comparative, developmental, and experimental studies have repeatedly demonstrated the invalidity of the nerve‐muscle specificity tenet since at least the beginning of the 20th century (Easter Jr. et al., 1985; Edgeworth, 1935; Harrison, 1904; Holmqvist, 1911; Luther, 1913; Shinohara, 1996; Straus Jr., 1946; Weiss & Hoag, 1946). Apparent violations of this axiom include, for example, wing muscles of bats innervated by the facial nerve (Thewissen & Babcock, 1991) and pelvic muscles of actinopterygians innervated by different spinal nerves depending on their position (jugular, thoracic, or abdominal) along the trunk (Winterbottom, 1974). The gular muscles of jawed fishes provide yet another example of a violation of the nerve‐muscle specificity principle. According to this tenet, muscles derived ontogenetically from the mandibular arch should be innervated exclusively by motor branches of the trigeminal nerve (cranial nerve V). In contrast, those derived from the hyoid arch should receive innervation solely from branches of the facial nerve (cranial nerve VII). If this were consistently true, the intermandibularis and its subdivisions would be innervated only by the trigeminal nerve, and the constrictor hyoideus ventralis and its derivatives only by the facial nerve. However, our analysis reveals that this idealized pattern is rarely observed among jawed fishes.

In most chondrichthyans, the facial nerve extends anteriorly and also innervates the intermandibularis (pers. obs; Luther, 1909a, 1909b). The trigeminal innervation of the intermandibularis is even completely lost in some lineages, as in hexanchiforms (pers. obs; Allis Jr., 1923; Holmqvist, 1911; Luther, 1909b). In contrast, actinistians and actinopterygians exhibit the opposite pattern: the trigeminal nerve extends posteriorly and innervates at least part of the interhyoideus (Figures 1, 3, 5, and 7). At the level of this muscle, branches of the trigeminal nerve commonly anastomose with those of the facial nerve (Figures 3, 5, and 7), indicating that both the hyohyoideus and intermandibularis may receive fibers from both cranial nerves. This pattern has been documented in the literature, and previous ichthyologists who have investigated the topic largely agree that disruptions in the trigeminal‐facial innervation pattern are ubiquitous in the gular muscles of actinopterygians (Holmqvist, 1911; Luther, 1913). Intraspecific and bilateral variations in the innervation of gular muscles have also been reported in both cartilaginous and bony fishes (Allis Jr., 1922, pp. 256, 283, fig. 50; Edgeworth, 1935, p. 47), indicating that innervation holds no greater phylogenetic or functional significance than other morphological attributes.

To be clear, we are not suggesting that innervation should be disregarded in assessments of muscle homology. On the contrary, for most cranial muscles, innervation patterns are remarkably conserved. Our point is that innervation should not serve as the sole criterion for accepting or rejecting a hypothesis of muscle homology. Instead, it should be considered alongside other attributes—such as origin, insertion, and position—as part of an integrative approach. For example, suppose we were to assume that the nerve‐muscle specificity paradigm holds true. In that case, part or all of the intermandibularis in chondrichthyans must have been lost (one evolutionary step). At the same time, the constrictor hyoideus ventralis would have expanded anteriorly, giving rise to a muscle with the same position, morphology, origin, and insertion as the intermandibularis in bony fishes (second evolutionary step). Conversely, in osteichthyans, part of the constrictor hyoideus ventralis would have been lost (third evolutionary step), while the intermandibularis would have expanded posteriorly to form a muscle identical in location, morphology, origin, and insertion to the constrictor hyoideus ventralis in cartilaginous fishes (fourth evolutionary step). Such a scenario would require twice as many evolutionary steps (the second and fourth being particularly improbable) as simply acknowledging that the facial nerve expanded its innervation anteriorly in chondrichthyans (one step), and that the trigeminal nerve expanded posteriorly in osteichthyans (second step). The nerve‐muscle specificity tenet not only contradicts parsimony but also runs counter to the very logic of evolutionary thinking. Evolution implies change over time. To propose that innervation is immutable is implicitly to suggest that it is not subject to evolution.

### 4.3 Functional inferences

The mandibular and hyoid muscles are responsible for feeding and respiratory movements in jawed fishes. These activities involve opening and closing the mouth, along with the associated expansion and contraction of the buccopharyngeal cavity. These muscles are typically activated at different times during feeding and breathing, allowing these activities to be divided into two to four main phases (Ballintijn & Hughes, 1965; Camp & Van Wassenbergh, 2025; Holzman et al., 2025; Lauder, 1980; Motta & Wilga, 2001; Osse, 1969). Regardless of differences in contraction timing, the mandibular and hyoid can be categorized into two types based on their primary action (Ballintijn & Hughes, 1965). The expansors open the mouth and expand the buccopharyngeal cavity, while the compressors close the mouth and contract the buccopharyngeal cavity (Datovo & Johnson, 2025).

Primitively in gnathostomes, the hypobranchial muscles − coracomandibularis (=branchiomandibularis) and coracohyoideus (=sternohyoideus, rectus cervicis) − along with the epaxialis and hypaxialis, are the main buccopharyngeal expansors (Bemis & Lauder, 1986; Lauder Jr., 1980; Lauder & Liem, 1980; Motta & Wilga, 2001; Osse, 1969; Schaeffer & Rosen, 1961). Most remaining mandibular and hyoid muscles, in turn, typically serve as buccopharyngeal compressors. Crown actinopterygians evolved additional active mechanisms of expansion of the oropharynx through changes in the attachment sites of the constrictor mandibularis dorsalis (Datovo & Johnson, 2025). The acquisition of insertions on the opercle (dilatator operculi) and on the lateral face of the palatoquadrate and hyomandibular (levator arcus palatini) transformed their primitive role as compressors into expansors.

A comparable chain of shifts in function is observed in some gular muscles in actinopterygians. In elasmobranchs, sarcopterygian fishes, polypteriforms, and acipenseriforms, the body of the interhyoideus is posteromedial to the hyoid bar (Figures 1 and 3). Therefore, contraction of its fibers approximates the distal regions of the hyoid bar, so the muscle acts as a compressor (Figures 2 and 4) (Bemis & Lauder, 1986; Lauder, 1980; Motta & Wilga, 2001). However, in neopterygians, the interhyoideus has an additional direct anterolateral connection with the lower jaw (Character 5), and most or all of its fibers lie lateral to the hyoid bar (Figures 5, 7, and 12). As a result, contraction of this muscle can also produce expansive movements, such as abduction of the hyoid bar or lower jaw. Electromyographic analyses confirm that the neopterygian interhyoideus may act either as a compressor or as an expansor, depending on the synchronous contraction of other cranial muscles (Figure 6) (Lauder, 1980; Lauder, 1983; Lauder & Liem, 1980; Osse, 1969). Similarly, attachment of the hyohyoideus inferior to the bases of certain branchiostegals, and the differentiation of the hyohyoideus abductor that occurs in some teleosts, presumably enable these muscles to perform expansive movements (expansion of the branchiostegal membrane). These modifications represent shifts from the primitive role of the hyohyoideus as a primary compressor of this membrane. Finally, it is worth noting that the primary actions of the gular muscles discussed here can be highly modified in derived teleost lineages, particularly those with specialized feeding habits (e.g., Lauder, 1983; Van Wassenbergh et al., 2014; Camp & Wassenbergh, 2025; Holzman et al., 2025).

The findings discussed above indicate that evolutionary innovations in the gular musculature that arose at basal nodes of the actinopterygian radiation expanded the mechanisms of buccopharyngeal expansion. Such modifications likely contributed to the predominance of suction feeding within the group. This feeding mode is widely regarded as a key factor in the evolutionary success of actinopterygians in aquatic environments, as it enables rapid and efficient prey capture over short distances (Lauder, 1980; Schaeffer & Rosen, 1961; Camp & Wassenbergh, 2025; Holzman et al., 2025).

## AUTHOR CONTRIBUTIONS

**Aléssio Datovo**: Conceptualization; data curation; formal analysis; funding acquisition; investigation; methodology; project administration; resources; software; supervision; validation; visualization; writing – original draft; writing – review & editing. **Paulo Presti**: Investigation; methodology; validation; visualization; writing – review & editing. **George Vita**: Investigation; validation; writing – original draft; writing – review & editing. **Murilo N. L. Pastana**: Validation; visualization; writing – review & editing. **Luiz A. W. Peixoto**: Data curation; validation; writing – original draft; writing – review & editing.

## Supporting information


**Data S1.** Script used for stochastic Bayesian reconstructions of ancestral states.

**Data S2.** Corrected Akaike Information Criterion (AICc) scores and Akaike weights (AICw) for equal‐rates (ER), symmetric (SYM), and all‐rates‐different (ARD) models for each character.

**Data S3.** Synonymy of terminology used for gular muscles in major previous studies.

## Data Availability

The data that support the findings of this study are available in the Supporting Information of this article.
